# Evaluation of sugar meal administered anti-*Leishmania* compounds on the vectorial capacity of the vector, *Lutzomyia longipalpis*

**DOI:** 10.1371/journal.pone.0325178

**Published:** 2025-06-25

**Authors:** Tainá Neves Ferreira, Samara Graciane Costa Latgé, Tadeu Diniz Ramos, Felipe Cerqueira Demidoff, Edézio Ferreira Cunha-Júnior, Paulo Roberto Ribeiro Costa, Marcus Vinícius Nora de Souza, Cláudia Regina Brandão Gomes, Viv Maureen Dillon, Chaquip Daher Netto, Eduardo Caio Torres Santos, Rod James Dillon, Mary Ann McDowell, Herbert Leonel de Matos Guedes, Fernando Ariel Genta

**Affiliations:** 1 Laboratório de Bioquímica e Fisiologia de Insetos, Instituto Oswaldo Cruz, FIOCRUZ, Rio de Janeiro, Brazil; 2 Laboratório de Imunobiotecnologia IMPG, Universidade Federal do Rio de Janeiro/ Laboratório de Imunologia Clínica, Instituto Oswaldo Cruz, Fiocruz, Rio de Janeiro, Brazil; 3 Laboratório Multiusuário de Pesquisa em Química, Instituto Multidisciplinar de Química, CM UFRJ-Macaé, Universidade Federal do Rio de Janeiro, Macaé, Brazil; 4 Laboratório de Imunoparasitologia, Unidade Integrada de Pesquisa em Produtos Bioativos e Biociências, Universidade Federal do Rio de Janeiro, UFRJ-Macaé Campus, Macaé, Brazil; 5 Laboratório de Química Bioorgânica, Instituto de Pesquisa em Produtos Naturais, Universidade Federal do Rio de Janeiro, Rio de Janeiro, Brazil; 6 Departamento de Síntese de Fármacos, Farmanguinhos, Fiocruz, Rio de Janeiro, Brazil; 7 Faculty of Health and Life Sciences, University of Liverpool, Liverpool, United Kingdom; 8 Laboratório de Bioquímica de Tripanosomatídeos, Instituto Oswaldo Cruz, Fiocruz, Rio de Janeiro, Brazil; 9 Biomedical and Life Sciences, Lancaster University, Lancaster, United Kingdom; 10 Eck Institute for Global Health, Department of Biological Sciences, University of Notre Dame, Notre Dame, Indiana, United States of America; 11 Instituto Nacional de Ciência e Tecnologia em Entomologia Molecular, Rio de Janeiro, Brazil; Federal University of Mato Grosso do Sul, BRAZIL

## Abstract

Multiple strategies involving the parasite-host-vector triad are necessary to control *leishmania*sis. One option is to prevent or reduce transmission of the pathogen by the phlebotomine sand fly vectors. In this sense, it is essential to explore compounds that may influence the vectorial capacity of the insect and reduce its longevity. We investigated the effect of anti-*Leishmania* drugs administered via the sugar meal on longevity, blood feeding, oviposition, and parasite load on the third day of infection of the sand flies, to identify the most promising candidates for vector infection tests. We identified compounds that affected the longevity of sand flies (three pterocarpanquinones – LQB-475, LQB-181, and LQ-03; one hydroxyethylpiperazine, PMIC-4, and Pentamidine), reduced oviposition of females after blood feeding (LQB-181 and PMIC-4), but did not decrease infection rates or parasite loads. The results provide the effect of antiparasitic drugs from the perspective of the insect vector.

## Introduction

Leishmaniasis is a group of diseases induced by protists of the genus *Leishmania,* transmitted via the bite of female phlebotomine sand flies [1–3]. It is listed as one of the top ten neglected tropical diseases, with more than 12 million people currently infected (0.9 to 1.6 million new cases yearly) and between 20,000 and 30,000 deaths per year [1,4]. This group of diseases is widely distributed in tropical and subtropical areas of the world [5].

*Leishmania* species are transmitted through the bite of female phlebotomines of the genera *Phlebotomus* in the Old World and *Lutzomyia* in the New World [4,6–8]. *Lu. longipalpis* is one of the main species of medical importance in Brazil for being the primary vector in VL and for its adaptive capacity in varied anthropic environments and permissiveness for different species of *Leishmania*, besides its typical, *Le. infantum* [8–12].

The *Leishmania* parasite has a digenetic life cycle, existing in two distinct forms: the amastigote form, which is rounded and intracellular, multiplying inside mammalian cells, particularly macrophages, and the promastigote, an extracellular, flagellated form found in the gut of sandflies [3,6,13,14].

Adult sand flies primarily feed on plant-derived sugars or aphid secretions, which serve as their primary sources of carbohydrates to meet their energetic requirements [8,10,15,16]. Females are also hematophagous and require blood to acquire the nutrients necessary for the development and maturation of their eggs. If the blood they ingest is infected, the parasites can be transmitted to another host during a subsequent blood meal [6,7,17,18]. The development of the parasite within the insect gut is crucial for transmission, as it undergoes developmental changes until reaching the infective metacyclic promastigote form. During this stage, the parasite remains in direct contact with the blood and sugar meals ingested by the insect.

Given the importance of the carbohydrate diet for sand flies, some groups have been investigating disease control strategies by using attractive sugar baits containing insecticidal components for controlling the insect vector [17,19–26]. The use of sugar-based toxic baits can be a valuable tool for delivering insecticidal compounds or antiparasitic agents inhibiting the parasite’s development within the insect vector’s midgut.

Recently, our group identified that secondary plant metabolites, added to the sugar solution, could reduce *L. mexicana* development in *Lu. longipalpis* [27]. The investigation of natural compounds impacting the development of *Leishmania* is a concept developed by Schlein and Jacobson [17,28,29]. By employing this innovative approach of delivering anti-*Leishmania* compounds through toxic sugar baits, this research has the potential to offer a more targeted strategy than conventional methods that use spraying of chemical insecticides, contributing to the ongoing efforts in combating this disease.

Overall, this research aims to identify compounds that could effectively control the spread of *leishmania*sis by targeting the insect vector and disrupting parasite development inside the insect by using a new strategy: delivering the compounds through toxic sugar baits. For this, we selected molecules that had an effect on promastigote forms of *Leishmania* sp. *in vitro*: two pterocarpanquinones (LQB-475, LQB-181), one furanonapthoquinone (LQ-03); one hydroxyethylpiperazine (PMIC-4), Pentamidine (Pent), and Amphotericin B (Amp). These compounds were used to evaluate the following aspects: the effects on adult longevity, influence on blood feeding (oviposition and quantity ingested), and the effect on the infection of *Lu. longipalpis* with *Le. amazonensis*. We designed this study as a proof-of-concept work for this series of compounds, choosing to target initially *Le. amazonensis* because it was used in previous studies and develops well in *Lu. longipalpis*, being a model already explored for the study of parasite-sand fly interactions [30]. Besides that, this parasite-sand fly association was already observed in the field, making *Lu. longipalpis* as a candidate for the transmission of cutaneous *Leishmania*sis in Brazil [31].

Antimonials have been the main drugs used in the treatment of patients with leishmaniasis for over a hundred years. The second line option is pentamidine, amphotericin B, miltefosine, or azithromycin [2,3,7,12]. However, these drugs have limitations such as toxicity or adverse effects. Another concern is the emergence of treatment-resistant strains of *Leishmania* [2,3,7,12,32,33].

In this context, many groups have been studying natural or synthetic compounds of analogous formulation to expand the arsenal of drugs available for leishmaniasis treatment. For example, chalcones, flavonoids, quinones, alkaloids, and terpenoids, which are phytoconstituents with anti-*Leishmania* activity and with the advantage of being environmentally acceptable [33–35]. Some compounds are also evaluated on the promastigote form of the parasite. Pterocarpanoquinones can be highlighted as synthetic compounds analogous to natural ones, which have shown promising results in *Leishmania* promastigotes and amastigotes [36–42].

The development of toxic sugar baits provides an opportunity to test the antiparasitic effect of these compounds within the insect vector. In this study, we evaluated the influence of different synthetic compounds with anti-*Leishmania* activity, including positive controls, amphotericin B and pentamidine, in different aspects of *Lu. longipalpis* physiology when added to the sugar diet. In previous publications, we found that these drugs affect the behavior of the vector insect when added to the sugar solution [27].

## Materials and methods

### Anti*-Leishmania* compounds

The drugs tested in this study were mostly modeled and synthesized at the Laboratory of Bioorganic Chemistry of the Nucleus for Research on Natural Products at the Federal University of Rio de Janeiro, Brazil [42]. They are synthetic compounds similar to those of natural origin with anti-promastigote action, mainly produced from naphthoquinones and pterocarpanes [37,41,42]. Pentamidine and amphotericin B, generally used in the treatment of leishmaniasis, were used as reference compounds. These compounds were received in solution in a stock concentration of 10 mM using dimethyl sulfoxide (DMSO) as solvent. Table 1 provides information on the compounds evaluated.

**Table 1:** Properties and experimental conditions of the anti-*Leishmania* compounds used in this work. The IC_50_ values represent half of the maximum inhibitory concentration for promastigotes of *Le. infantum* or *Le. amazonensis*. The concentration used in the experiments with LQB-475 and Pent was 10 times the value of the higher IC_50_observed for both *Leishmania* species. For LQB-181, PMIC-4, and Amp the *Le. amazonensis* IC_50_ was used, and for LQ-3 the *Le. infantum* IC_50_ was used_._ N.D.: Not determined. Structures of compounds are presented in Supplementary Table 1.

Compound	Class	IC_50_ *Le. infantum/Le. amazonensis* (μ mol L^ − 1^)	Concentration (μ mol L ^−1^)	Reference
LQB-475	Pterocarpan-quinone	1.4/ 0.4	14.0	[41]
LQB-181	Pterocarpan-quinone	N.D./ 1.98	20.0	[43]
LQ-03	Pterocarpan-quinone	1.7/ N.D.	17.0	[44]; this work
PMIC-4	Hydroxyethyl-piperazine	N.D./ 1.8	18.0	[45,46]
Pentamidine (Pent)	Aromatic-diamidine	5.7/ 4.8	57.0	[41]
Amphotericin B (Amp)	Polyene	N.D./ 2.2	22.0	This work
DMSO	Solvent	N.D./ N.D.	0.57%	–

For anti-*Leishmania* activity, promastigotes of *Leishmania amazonensis* were adjusted to a concentration of 1 × 10^6^ cells/mL in Schneider medium supplemented with 10% of fetal bovine serum and incubated at 26 °C for 72 h with Amphotericin B or LQ-03 (0–10 µM). The antileishmanial activity was evaluated by adding in each well 22 μL of MTT [3-(4,5-dimethylthiazol-2-yl)-2,5-diphenyltetrazolium bromide] at 5 mg/mL (Sigma®). After 2 h, 80 μL of DMSO was added into each well to solubilize the formazan crystals. The optical density was determined at a wavelength of 570 nm in microplate reader (µQuant Bio-Tek Instruments®, Winooski, state, USA). Logarithm regression analysis was performed using GraphPad Prism 5.0 (San Diego, CA, USA) to obtain the values of IC50.

### Preparation of sugar solution containing compounds

The sugar solution used in all experiments was prepared with commercial sucrose (Sigma) in a concentration of 70% (w/v), sterilized in an autoclave, and refrigerated at 4ºC. Compounds LQB-475, LQB-181, LQ-03, PMIC-4, Pentamidine (Pent), Amphotericin B (Amp) (10 mM stock solutions in DMSO) were added to the sucrose 70% (w/v) solution to achieve a final concentration corresponding to 10x the value of the selected IC_50_ for *Leishmania* sp. (see Table 1 for details). For the experiments, two control groups were used: Control 1 was a sucrose 70% (w/v) solution, and Control 2 (referred to as DMSO) was a solution of DMSO 5.7% (v/v) in the sucrose 70% (w/v) solution. In all experiments, the sucrose solution was offered to the insects in 20 drops of 5 µL each, on top of a petri dish covered with parafilm [47]. See Supplementary Table 1 for details.

### Effect of compounds on the longevity of sand flies

Insects used in all experiments were from the colony of *Lu. longipalpis* of the Laboratory of Insect Biochemistry and Physiology of the Oswaldo Cruz Institute, established from insects collected in Jacobina (Bahia state, Brazil). The colony was kept according to Refs 16 and 27.

Experiments were performed with 20 newly emerged adults per cage (males or females, separated). For each cage, either the sucrose solution containing the compounds, or only sucrose 70% (w/v) (control 1), or 5.7% (v/v) DMSO in sucrose solution (control 2) was offered to the insects as described above [47]. The sugar solution was changed every two days, and the stocks were kept at −20 ºC. The mortality was observed daily. Insects were kept in an incubator under controlled conditions (26 ºC and 70% humidity), according to previously described studies [27,48]. In accordance with our previous reports [47], all the sand flies fed on the sugar solutions in these conditions. The experiments were carried out in triplicate.

### Effects of compounds on blood feeding and oviposition

#### Blood feeding.

Cages (n = 7) containing about 30 females and 30 males, 1–2 days after emergence, were used in this experiment. As described above, the sucrose solution containing compounds, or the control (sucrose only or DMSO) was offered to the insects in each cage. The solutions were changed every two days, and the stocks were kept in a freezer (−20 ºC). After seven days, the females were offered an anesthetized hamster for 30 minutes (License CEUA/IOC – L-029/2016). After the feeding, the engorged females were counted, and these insects were separated and dissected on the same day to observe the gut and quantify the ingested blood. The quantification was performed through protein quantification using the bicinchoninic acid method (PierceTM et al. - Thermo Fisher Scientific) [49], following the instructions provided by the manufacturer for microplate procedures, using bovine serum albumin as a standard [50]. The amount of protein originated from insect tissues was considered to be a minor component of the sample, and total protein measurements were assumed to reflect the nutritional intake of each insect.

#### Fecundity and Fertility.

The female mortality was evaluated before blood feeding (7 days of contact with compounds) and three days after post-blood feeding. Around two hours after feeding, blood-fed females were separated into small plastic containers containing plaster in the bottom to maintain humidity. These containers create a favorable environment for the females to lay eggs. Aspects related to oviposition were evaluated, including the number of eggs, fecundity (eggs/female), and fertility (whether the eggs hatched or not), which were monitored up to 14 days after blood feeding. The number of eggs laid was quantified by visually counting the eggs in a photo taken in a stereoscope (Carl Zeiss – Stemi DV4, Werk Gottingen, Germany). Editing tools such as Photoscape (v3.7), the ImageJ program (1.53e), and image enlargement (zoom) were utilized. In ImageJ, the same image underwent an editing process involving 8-bit conversion, with threshold adjustment, black and white scale, selection of the area of interest, and particle analysis, as recommended by the program. The visual counting and ImageJ quantification data were used to calculate an average with the standard deviation of the average (SEM). No eggs were observed on the sides or in the top of the oviposition pots.

For compound LBQ-181, it was necessary to increase the initial number of females per replicate from 30 (replicate 1) to an average of 50 (replicates 2–5). Controls (sucrose and DMSO) and PMIC-4 had an average of 30 females (5 replicates). For the other compounds, 3 replicates with 30 insects each were performed.

### Effect of compounds on *Lu. longipalpis* infection with *Leishmania*

#### Animal handling.

Mice aged 12–16 weeks were infected with *Le. amazonensis* (JOSEFA strain) by direct injection containing 2 × 10^6^ parasites mL^-1^. The infection of the insects was done 70 days after mouse infection when the paws contained a high parasite load. The infection process with *Le. amazonensis* simulates a natural infection, where female sand flies fed directly from the infected foot of Balb/c mice. To facilitate blood feeding, Balb/c mice were anesthetized with xylazine (10 mg/kg) plus ketamine (100 mg/kg) before exposure to the sand flies (Ethic Committee License Numbers L-029/2016 CEUA-IOC, and 080−18 UFRJ).

#### Sand fly infections.

In this experiment, only the most promising compounds, with significant results in the previous test, were used to evaluate the effect on *Lu. longipalpis* infection with *Le. amazonensis*. The sucrose solution containing compounds LQB-475, LQ-03, Pent, and DMSO were used. Another control group was included, with insects feeding only on sucrose 70% (w/v) before and after blood feeding.

For infections, about 200 females 1–2 days after emergence were separated into fabric cages. Insects were fed for two days on a control diet (sucrose 70% (w/v)) or a test diet (sucrose plus compound or DMSO) before the infective feeding. Mice were placed outside the top part of the cage, standing over a plastic cover to prevent insects from biting other portions of the mice. The insects accessed the infected foot through a hole in the top of the cage. Blood feeding was conducted for 75 min, and two mice were used for each cage. After feeding, the mice were removed from the top of the cage and euthanized. This experiment was repeated two to three times.

#### Leishmania countings.

After blood feeding, only the engorged females were kept in the cage and maintained on the same sugar diet as previously established (control – sucrose or test – sucrose plus compound or DMSO), according to described above. For analyzing the infection, 10–15 samples were collected on the 3^rd^, 5^th^, and 7^th^ days after blood feeding in each repetition, corresponding to the different stages of evolution of the *Le. amazonensis* infection in *Lu. longipalpis***.** Each gut was dissected and gently homogenized in 10 μL of PBS, and quantification was performed with the aid of a hemocytometer.

### Statistical analysis

Data was analyzed using GraphPad software Prism (version 6) for Windows. The D’Agostino and Pearson test was used to verify the normal distribution of the data. Data with normal distribution were submitted to the unpaired t-test or Analysis of Variance (One-way ANOVA) with Tukey’s multiple comparison post-test. Non-normally distributed data were analyzed trough the nonparametric Mann-Whitney or Kruskal-Wallis test with Dunn’s multiple comparison post-test. For survival, results were analyzed using the Kaplan-Meier survival curve, and the median survival time was determined in each condition. The log-rank Mantel-Cox test was used to compare survival curves, and Dunn’s multiple comparison post-test was used to compare tree survival replicates’ mean and median values. All comparisons with *p-value *< 0.05 were considered statistically significant.

## Results

### Effect of compounds on the longevity of sand flies

**Fig 1:**
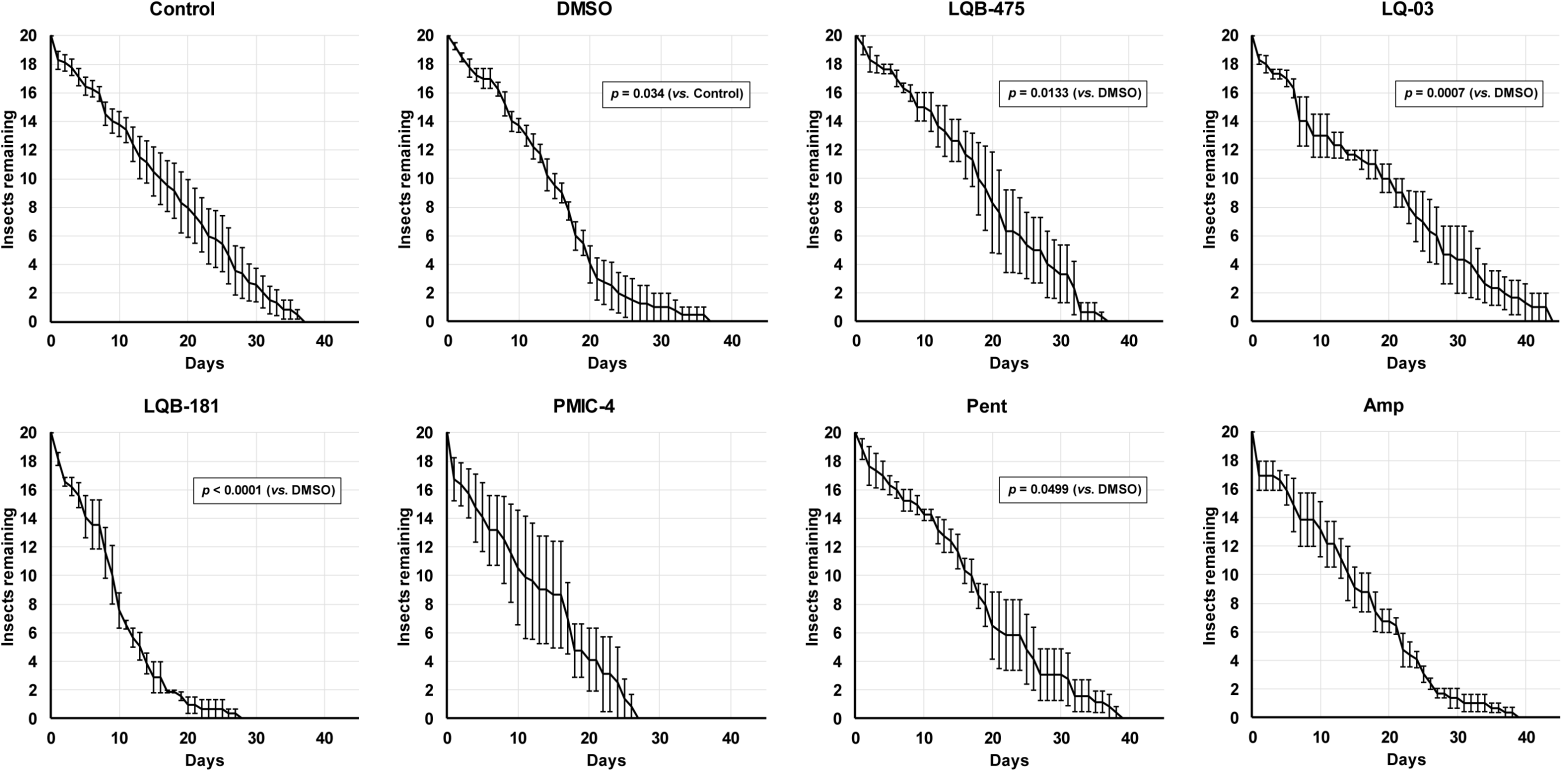
Effect of anti-*Leishmania* drugs added to a sugar diet on the longevity of *Lu. longipalpis* males. Insects were kept under controlled conditions (26 ºC and 70% humidity), and mortality was checked daily. In the control group, insects were fed with a solution containing only sucrose 70% (w/v). All results are the mean ± SEM. Comparison test between log-rank survival curves (Mantel-Cox). Experiments were performed at least three times independently, with n = 20 each. See Material and Methods and Table 2 for details.

We analyzed the impact of the anti-*Leishmania* compounds on the longevity of both male and female sand flies using sucrose diets. Because the stock solutions of the compounds were prepared in DMSO, we included a control group fed on sucrose with DMSO, and all the statistical comparisons were performed against this group. However, because we were also concerned about the physiological impact of DMSO, we also performed a comparison of insects fed with DMSO against insects fed with sucrose only. Importantly, addition of DMSO has a significant impact in the longevity of males when compared to insects fed only with sucrose (Fig. 1, p = 0.034, Log-rank test). The longevity of insects fed with the LQB-181 compound was significantly shorter than the longevity of insects fed with sucrose plus DMSO (Log-rank test, *p *< 0.0001). Compounds LQ-03 (Log-rank test, *p *< 0.001), LQB-475 (Log-rank test, *p* < 0.05), and Pent (Log-rank test, *p *< 0.05; Fig 1) also resulted in longevities significantly different to the longevity of insects fed with sucrose plus DMSO. The LQB-181 compound shortened the average lifespan (ALS) to 10 ± 1 days, while DMSO led to an ALS of 15 ± 1 days. An opposite effect was observed in ALS of males fed on Pent, LQB-475, and LQ-03, which increased the ALS respectively to 17 ± 1, 18 ± 1, and 19 ± 2 days (Table 2).

**Table 2:** Median and average lifespans (ALS) of *Lu. longipalpis* feeding on sugar baits containing anti-*Leishmania* compounds. Male and female controls were fed only the sucrose diet (Ctrl). These values were obtained by survival analyses and *p* values were calculated by Log-rank survival curves (Mantel-Cox) comparative test for males: Ctrl/DMSO – *p* = 0.034, LQB-475/DMSO = 0.0133, LQ-03/DMSO = 0.0007, LQB-181/DMSO < 0.0001, and Pent/DMSO = 0.0499, and females: LQB-475/DMSO = 0.0048, and PMIC-4/DMSO = 0.0032. ALS was calculated considering the lifespan of all individuals.

	males							
	**Ctrl**	**DMSO**	**LQB-475**	**LQ-03**	**LQB-181**	**PMIC-4**	**Pent**	**Amp**
median	17	15	19	20	10	13	18	15
ALS ± SEM	17 ± 1	15 ± 1*	18 ± 1*	19 ± 2***	10 ± 1****	13 ± 1	17 ± 1*	15 ± 1
n	120	80	60	60	60	60	60	60
	**FEMALES**							
	**Ctrl**	**DMSO**	**LQB-475**	**LQ-03**	**LQB-181**	**PMIC-4**	**Pent**	**Amp**
median	12	15	8	12	13	12	19	17
ALS ± SEM	14 ± 1	16 ± 1	11 ± 1**	13 ± 1	16 ± 1	11 ± 1**	19 ± 1	17 ± 1
n	100	60	60	60	60	60	60	60

**Fig 2:**
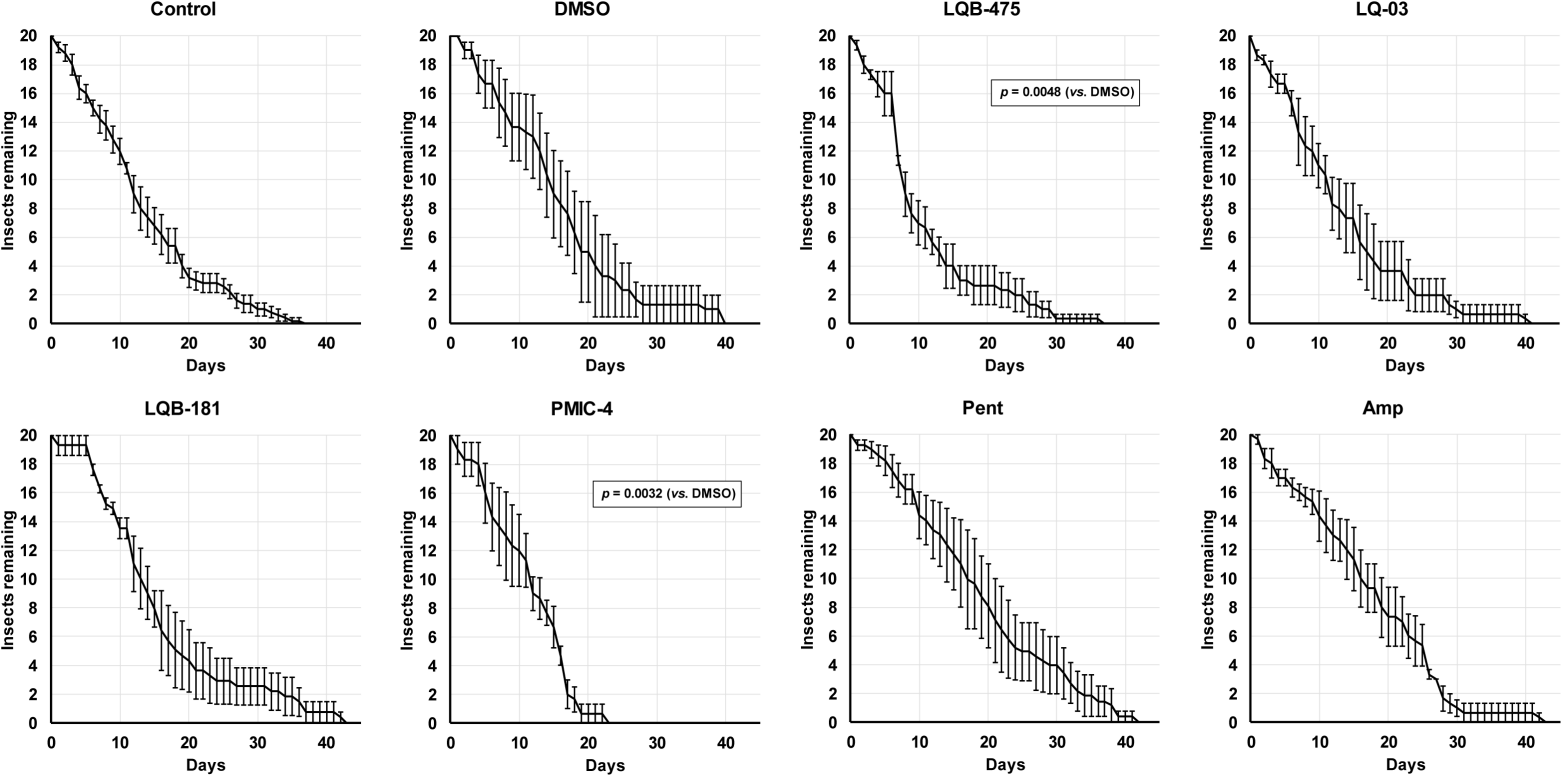
Effect of anti-*Leishmania* drugs added to a sugar diet on the longevity of *Lu. longipalpis* females. Insects were kept under controlled conditions (26 ºC and 70% humidity), and mortality was checked daily. In the control group insects were fed with sucrose 70% (w/v). All results are the mean ± SEM. Comparison test between log-rank survival curves (Mantel-Cox). Experiments were performed at least three times independently, with n = 20 each. See Material and Methods and Table 2 for details.

Interestingly, adding DMSO to the sugar did not affect significantly the longevity of females (Fig 2, *p* > 0.05, Log-rank test). The longevities of females fed on LQB-475 (Log-rank, *p *< 0.01), and PMIC-4 (Log-rank, *p *< 0.01) (Fig 2), were significantly lower to the longevity of females feeding on sucrose containing DMSO. We detected a reduction in ALS when comparing LQB-475 and PMIC-4 groups *vs*. the DMSO group, from 16 ± 1 days to 11 ± 1 (Table 2).

### Effects of anti-*Leishmania* compounds on blood feeding and oviposition

**Fig 3:**
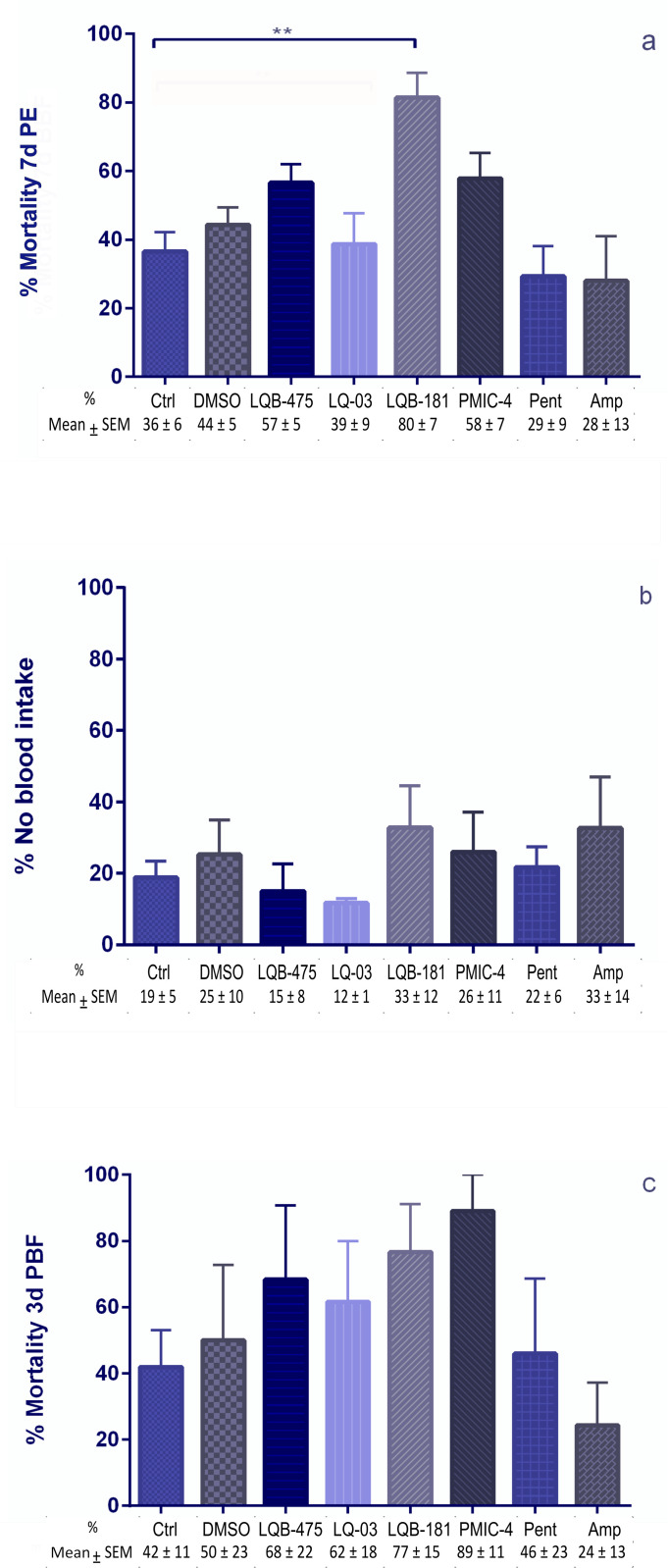
Effect of anti-*Leishmania* compounds added to a sugar diet on females of *Lu. longipalpis* pre and post-blood feeding. **(a)** Mortality of females before blood feeding. These females were offered the anti-*Leishmania* compound in a sugar diet for seven days before blood feeding. 7d PE: 7 days post-emergence. **(b)** Percentage of females that are not engorged after blood feeding. Females were exposed to the source of blood for 30 min. **(c)** Mortality of females three days after blood feeding. Females continued to be offered anti-*Leishmania* compounds in the sugar diet after blood feeding. 3d PBF: 3 days post-blood feeding. All percentage results are the mean ± SEM. For LQB-181, 5 replicates were performed, 1 with 30 insects and 4 with 50 insects each. For Controls and PMIC-4, 5 replicates with 30 insects each, and for the remaining compounds, 3 replicates with 30 insects each were performed. Dunn’s multiple comparison test, ** *p *= 0.007.

**Fig 4:**
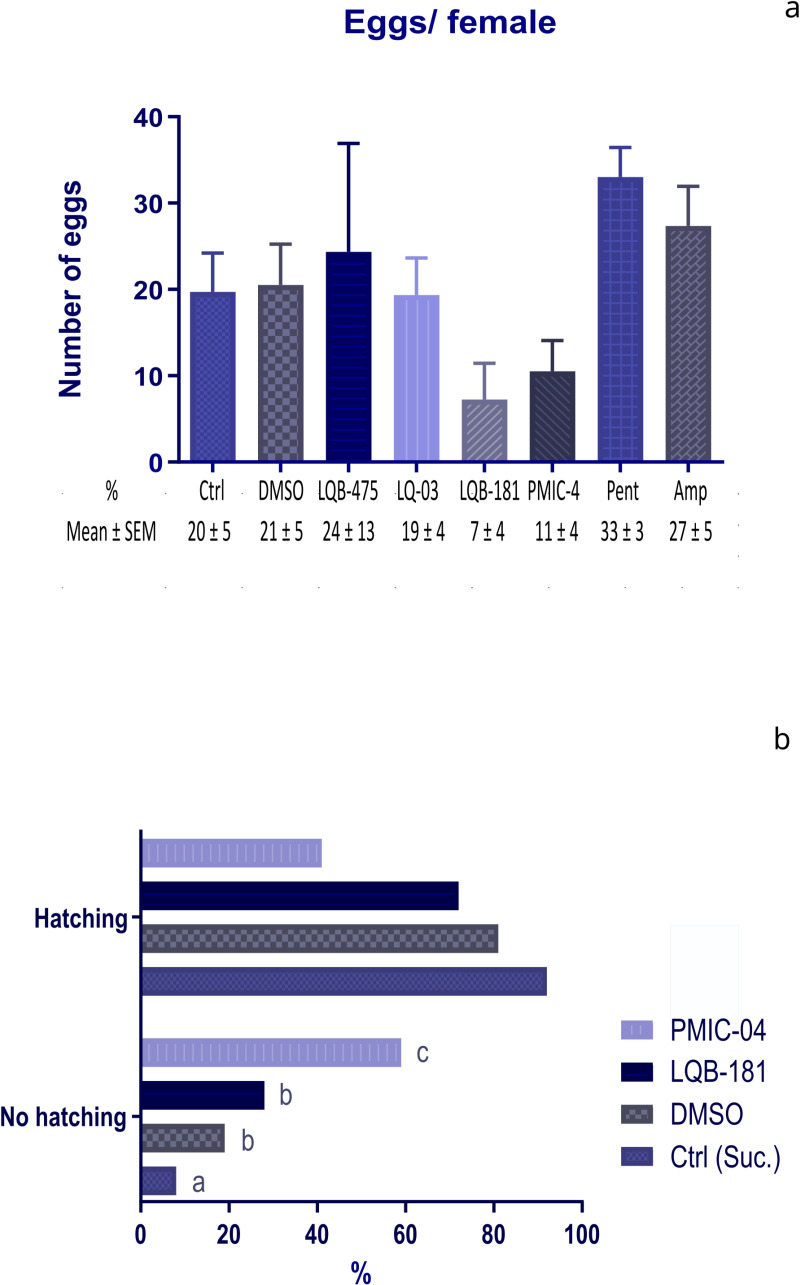
Effect of anti-*Leishmania* compounds on fecundity of females and egg fertility. The compounds were added to a sugar diet and offered to females seven days before blood feeding and three days after when females were separated for oviposition. **(a)** Fecundity (eggs laid per female). The number of eggs was accessed seven days after oviposition. **(b)** Fertility (whether the eggs hatched or not). The hatching was evaluated 14 days after oviposition. The percentage results consider the total number of eggs hatching or not in each replicate (three to four repetitions). Different letters beside the bars indicate groups that are statistically different from each other, according to Fisher’s exact test (*p* < 0.05). For DMSO and PMIC-4, 4 replicates with 30 insects were performed. For LQB-181, 4 replicates with 50 insects were performed. For the other compounds, 3 replicates with 30 insects each were performed.

Besides evaluating the effect of compounds on the longevity of insects, we also analyzed how these compounds affect female blood feeding and their impact on other aspects like fecundity and fertility. Evaluating the effects of the drugs added to a sugar diet before a blood meal, we did not see significant differences in mortalities when comparing the group fed with sugar plus DMSO to the insects fed with sugar only. LQB-181 caused higher mortality in females and reduced the number of insects available for blood feeding when comparing to sugar-fed controls. However, there was no significant difference when comparing the insects treated with LBQ-181 to the insects exposed to sugar containing DMSO (Fig 3a). No statistical difference was detected in the number of engorged females (Fig 3b) or survival of females three days after blood feeding (Fig 3c) between any of the groups considered. Although there is a reduction tendency caused by LQB-181 and PMIC-4 in the female fecundity, no statistical differences were noticed between all groups tested (Fig 4a).

We assessed the viability of the eggs, by observing the hatching and if these larvae were alive and active. For this aspect, we checked the hatching rate of the eggs in each group tested. The group fed with sugar plus DMSO had a hatching rate significantly lower than the group fed with sucrose only (Fisher’s exact test, *p *= 0.037). There was no significant difference between the treatments with LQB-181 and DMSO (Fisher’s exact test, *p* = 0.567), but the groups treated with PMIC-4 and DMSO showed significant differences to each other (Fisher’s exact test, p < 0.0001). Fig 4b illustrates the hatching percentages for each compound.

Five replicates were performed to test the effect of the LQB-181 compound. As previously demonstrated, the compound caused a high mortality of females after seven days of feeding (sucrose + compound) before blood feeding. In these experimental replicas, one did not have enough females for oviposition (these samples were directed to dissection and protein quantification), and the females laid no egg in the other two. Females laid eggs in the other two replicates, but only in one of the two experimental replicas did the eggs hatch days later. That means that 36% of the total number of females (4/11) separated for oviposition did not lay eggs.

For tests with compound PMIC-4, of the four experimental replicates performed in which we had females for oviposition, females did not lay eggs in one of the replicas, corresponding to 26% of total number of females (4/15). In the other three replicas, even with posture, the eggs hatched only in one of the replicas.

**Fig 5:**
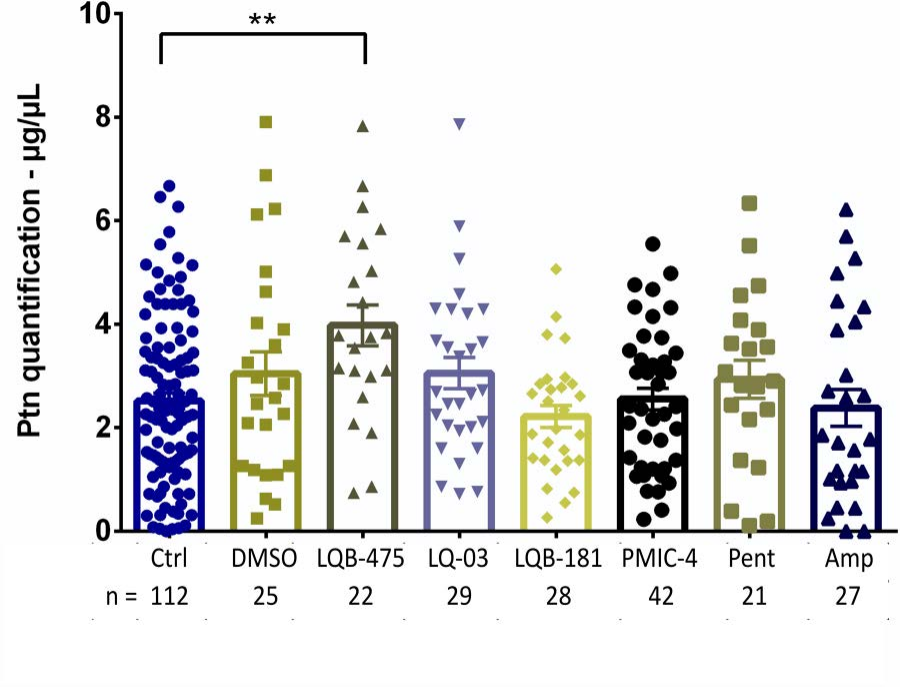
Quantification of ingested proteins by females after 30 minutes of blood feeding. Females were previously fed with a sugar solution containing anti-*Leishmania* compounds for seven days. Proteins were quantified from the blood present in the intestinal contents of females immediately after blood feeding, using the BCA method. The percentage results are the mean ± SEM of three to five (LQB-181 and PMIC-4) biological replicates. Dunn’s multiple comparisons test, **p < 0.01. Each symbol (square, triangle, diamond or circle) shows the measurement from one individual insect.

We investigated whether adding these compounds to the sugar diet before blood feeding could inhibit the amount of ingested blood. For this, we quantified the proteins in the gut content of engorged females. Adding DMSO to the sugar did not affect the amount of blood ingested when compared to insects previously fed with sugar only. None of the compounds resulted in significantly different amounts of blood ingested when compared to the insects fed with sugar plus DMSO. The average amount of protein ingested by the females was between 2.2 ± 0.2 and 4.0 ± 0.4 µg/µL. Fig 5 shows the result of these quantifications.

### Effect of compounds on *Lu. longipalpis* infection with *Le. amazonensis*

To determine the effect of compounds added to the sugar diet on the infection of *Lu. longipalpis* with *Le. amazonensis,* the compounds LQB-475, LQ-03, and Pent were selected. This was based on the *in vitro* effect of the compounds over promastigote forms and on the impact on insect physiology described in this study and the previous study published by our group [47]. Compounds LQB-181 and PMIC-4 were not used for this further evaluation. Compound LQB-181 affected the longevity of females before blood feeding (Fig 3a), which limits the number of females available for infective blood feed and the assessment of the course of infection. Compound PMIC-4 affected the longevity of males and females (Table 2; Figs 1 and 2) and has more pronounced effects related to oviposition.

**Fig 6:**
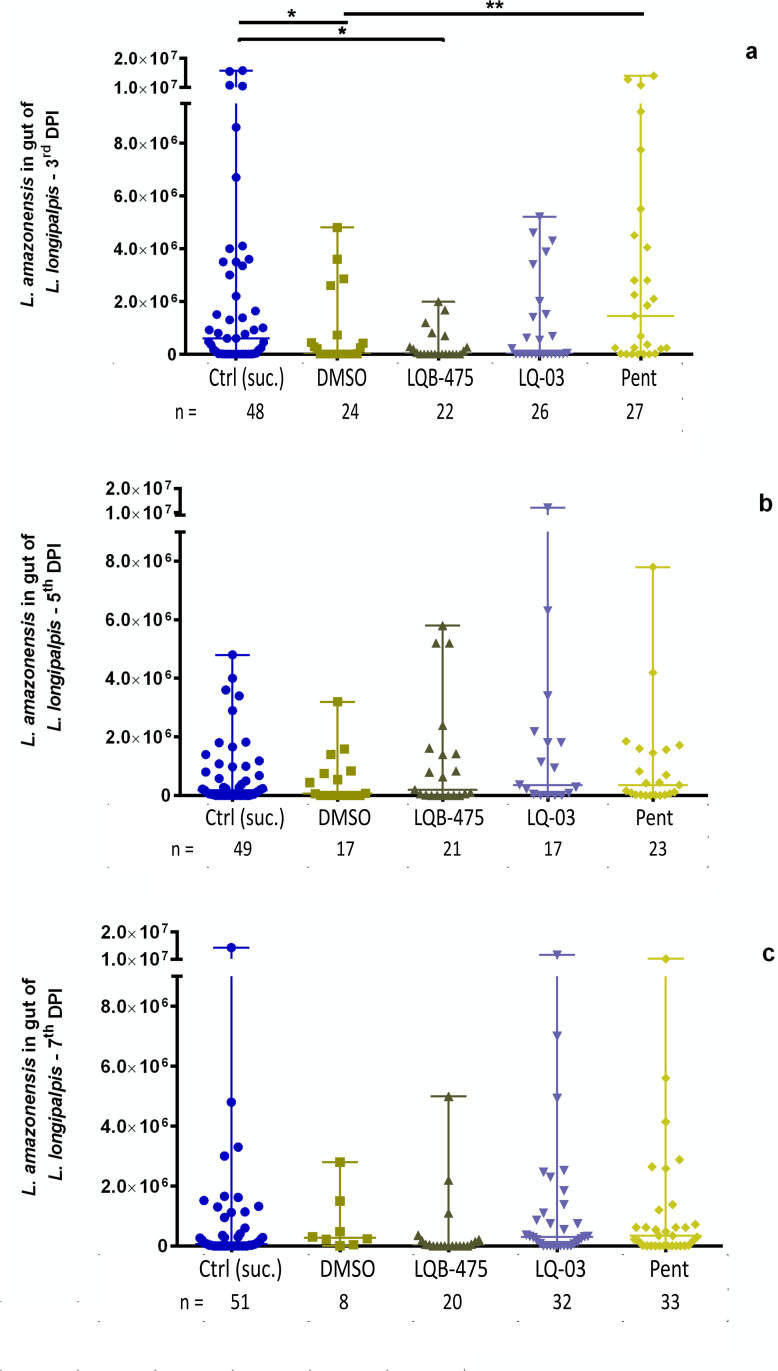
Influence of anti-*Leishmania* compounds added to the sugar diet on the infection of *Lu. longipalpis* with *Le. amazonensis.* Parasite counts were measured on the 3rd, 5th, and 7th day after infection. Significant differences were investigated by Dunn’s multiple comparisons test and Mann-Whitney test.

Of the infections carried out with *Le. amazonensis*, we observed that insects fed with sugar plus DMSO had significantly lower parasite counts than the controls fed with sugar only, in the third-day post infection (PI) (*p* = 0.0108, Mann-Whitney test) (Fig 6). Interestingly, on this day insects fed with Pent showed higher parasite counts than the insects treated with DMSO (*p* = 0.0048, Mann-Whitney test, Fig. 6). However, the progression of infection was not affected by any of the compounds tested, since any differences between all groups were not observed on the fifth- or seventh-day PI (Fig 6).

In terms of the percentage of infected (positive) females compared with the total number of insects dissected in the three days analyzed, we observed a trend similar to what was seen on parasite counts. On the third day PI, insects fed with sugar plus DMSO showed a significant lower prevalence of infection (46%) when compared to controls fed with sugar only (75%) (*p* = 0.0192, Fisher’s exact test, Table 3). Besides that, on the same day the insects treated with Pent showed a prevalence of infection (81%) significantly higher than the insects fed with sugar plus DMSO (46%) (*p* = 0.01, Fisher’s exact test, Table 3). On the 5^th^ and 7^th^ after infection, no significant differences were observed between any of the groups (Table 3). Table 4 summarizes all results obtained for the compounds described in this work.

**Table 3:** Effect of compounds added to the sugar diet on the prevalence of infection of *Lu. Longipalpis* with *Le. amazonensis*. Numbers and percentages of females infected with *Le. amazonensis* over the post-infection days.

	Ctrl (Suc.)	DMSO	LQB-475	LQ-03	Pent
Ni/ Nt	36/ 48	11/ 24	10/ 22	13/ 26	22/ 27
3 DPI	75	46	48	50	81
Ni/ Nt	33/ 49	9/ 17	15/ 21	13/ 17	19/ 23
5 DPI	67	53	71	76	83 ± 1%
Ni/ Nt	32/ 51	7/ 8	10/ 20	23/ 32	23/ 33
7 DPI	63	88*	50	72	770

Abbreviations: Ni = number of infected females; Nt = total number of females analyzed; DPI = days post infection.

**Table 4:** Summary of the effects of antiparasitic drugs on the physiology of *Lu. longipalpis.* Summary of the data on longevity (males and females), mortality of females before blood feeding, egg hatching, blood intake, and infection with *Le. amazonensis.*

	DMSO	LQB-475	LQB-181	LQ-03	PMIC-4	Pent	Amp
**Longevity**	↓♂	↑♂	↓↓↓♂	↑♂		↑♂	—
	—	↓↓↓♀	—	—	↓↓↓♀		—
**Mortality before blood-feeding**	—	—	—	—	—	—	—
**Hatching of eggs**	81%	100%	72%	100%	41%	100%	100%
**Blood intake**	—	—	—	—	—	—	—
**Parasitic load *Leishmania* amazonensis**	↓ (3 DPI)	—	n.d.	—	n.d.	↑ (3DPI)	n.d.
**% Infection**	↓ (3 DPI)	—	n.d.	—	n.d.	↑ (3DPI)	n.d.

Abbreviations — = no significant change compared to controls with a diet containing only sucrose 70% (w/v); n.d. = not determined; ↓ = reduction or ↑ = increase of the analyzed parameter; DPI = days post infection.

## Discussion

In this study, we added compounds to the sugar diets to examine the effects of antiparasitic drugs on some aspects of the biology of sand flies by adding these substances to the sugar diet. Sugar baits are a well stablished strategy to reduce insect populations and can be a new approach to reduce their vectorial capacity. In this context, we must consider a new perspective as treating insects with antiparasitic drugs, using sugar baits to prevent infection. In this respect, it is strategic to select for compounds with no anti-feeding effect. We evaluated the effect of selected compounds on the longevity of both female and male sand flies, the effect on blood feeding and oviposition, and lastly, the infection of *Lu. longipalpis* with *Le. amazonensis*. The compounds tested were chosen based on their known activity against *Leishmania*, and in the absence of repellent and anti-feeding activity on sugar meals [47]. The characterization of the effects of the ingestion of these compounds in sugar meals can help in the development of a new generation of transmission-blocking baits, aiming at the parasite, vectorial capacity, and other aspects of vector fitness, like blood feeding or reproduction.

The first aspect evaluated was the effect of the compounds on the longevity of insects. Of the compounds tested, only the Amphotericin B (Amp) did not affect longevity in males or females. Compounds LQB-181 and the solvent DMSO caused a reduction in ALS in males, and compounds LQB-475, LQ-03, and Pent unexpectedly increased lifespan. For females, LQB-475 and PMIC-4 caused a decrease in ALS. Three of the compounds tested are classified as pterocarpanoquinones (LQB-475, LQB-181, and LQ-03), and compound PMIC-4, which exhibited a longevity-reducing effect in females, is a hydroxyethylpiperazine derivative. These results are promising since we are not using high concentrations of these substances (between 14 and 57 μmol. L^-1^) to evaluate the possibility of using them in baits for insect control. The sugar bait containing Amphotericin B (Amp) (924.09 g·mol^-1^), for example, had only 0.002% of the antiparasitic compound (22 µmol. L^-1^), a concentration at least twenty times lower than the 0.05 to 0.25% concentration of active compound in insect control commercial products [51].

Some studies have demonstrated the effect of quinones on the mortality and feeding of insects from different orders as Diptera, Isoptera and Coleoptera [52–58].. The possible mechanism of these compounds involves damage the midgut after digestion, and inhibition of the mitochondrial complex III. Quinones, known as maesanin and juglone, possibly affect the energy generation mechanism in insects by inhibiting the mitochondrial electron transport chain [59]. These effects may explain why the reduction in longevity in sand flies is not immediate but rather a long-term effect.

Furthermore, this fact agrees with another capacity of quinones, such as naphthoquinones, that are redox active and can generate oxidative stress mainly in oxygen-rich environments [60,61]. These substances can also generate anti-feed or toxic effects due to their ability to alkylate essential nutrients and cellular components [60]. Inhibitory feeding effects were also observed in pterocarpanes extracted from *Pterocarpus macrocarpus* in tests carried out with *Spodoptera litura* [62].

The unexpected increase in ALS caused by LQ-03 and LQB-475 in males could be related to a structural detail in this pterocarpanquinone, an ortho-quinone. Molecules with this configuration are involved in sclerotization and cuticle darkening. This process occurs by reaction of a protein or an amino acid with a secreted polyphenolic derivative in the presence of an oxidase. Tissue hardness and stiffness involve the oxidation of these compounds to ortho-quinones primarily, followed by condensation with the NH_2_ (or still SH) groups of proteins [63,64]. The sclerotization can also promote the darkening of the cuticular color (brown/black color), which favors the absorption of solar radiation, keeping the insects warm [64,65]. However, further studies would need to be conducted to assess whether LQ-03 and LQB-475 would benefit the cuticular structure of adult sand flies.

Of those compounds, LQB-181 generated the most significant effects in reducing male longevity. Structurally different from other quinone derivatives, this compound has an amide group linking oxygen, suggesting that this nitrogenous portion might be responsible for enhancing the effect in males. Previous results demonstrated an anti-feeding effect of compound LQB-181. When offered a choice, males prefer to feed on a diet containing solely sucrose (54% vs 33%). Besides that, males fed on the sugar bait containing LQB-181 compound ingested the same volume as males fed on a sucrose diet [45]. The results demonstrate that even though LBQ-181 presented a toxic effect, males fed on a diet containing this drug due to a lack of another source of sugar.

PMIC-4, a hydroxyethyl piperazine derivative, caused a reduction in ALS in females and showed promise for use as an insecticidal compound for sand flies. Previously, PMIC-4 was ignored by males when there was a choice between diets [47], and even the volume of solution ingested was reduced compared to the control diet. Similarly to LQB-181, males ingested a smaller amount of PMIC-4, but since they had no other diet option it increased their mortality. Literature data show that piperazine-derived synthetic compounds exhibit larvicidal or growth inhibitory effects on caterpillars and mosquito larvae [66–68].

Compounds LQ-03 and Pent (pentamidine) caused an increase in the average lifespan of males. When looking at preference and ingestion results [47], we did not detect preference or higher ingestion in solutions containing these compounds. Pent seems to be palatable for sand flies since, in the experiments of ingestion of the sugar diet, the ingested volume of the pentamidine solution (68 ± 10 nL) was one of the highest compared to the other diets. These results suggest that Pent was beneficial for the insect’s fitness.

These data highlight the potential of these drugs to influence the epidemiology of the disease, especially those related to pentamidine, which is already one of the reference drugs used to treat leishmaniasis [2,3]. In this work, we verified the influence of this compound via the sugar diet; nonetheless, it would be relevant to assess whether an increase in longevity would also occur in a female that obtained a blood meal from a vertebrate host, e.g., a dog, being treated with this drug. In this scenario, we would have a potentially infected female with an increased lifespan and higher chances of making new blood feeds and spreading the pathogen. Our results infecting sand flies with *Le. amazonensis* also demonstrated that the insect’s ingestion of pentamidine does not inhibit the parasite’s development. All this reinforces the non-recommendation of treating dogs with the same drugs used to treat humans. Besides that, medications such as amphotericin B and pentamidine isothionate seem to have low efficacy in treating dogs and increase the risk of selecting parasites resistant to these drugs [2].

It is also worth mentioning that although compounds LQB-181 and PMIC-4 did not cause a statistically significant reduction in the number of eggs/females (Fig 4a), they seem to influence the laying and hatching of eggs (Fig 4b). These results reinforce that LQB-181 and PMIC-4 were the most deleterious compounds for the physiology of sand flies, suggesting that digestion or alterations in digestive products could affect egg production. These results can be incorporated into future studies to assess whether these effects would persist at different concentrations or experimental designs.

It is important to highlight, in the context of future applications of the compounds above for sand fly control, that LQB-475 has low toxicity against mammalian cells, with selective indexes of 23.76, 5.53, and >40 for *Le. amazonensis*, *Le. brasiliensis*, and *Le. infantum*, respectively [39]. Besides that, LQB-181 was shown to be selective for cancer cells and *Le. amazonensis*, with selective indexes of 13.7 and 2.8, respectively [43]. PMIC-4 was shown to be safe in a murine model, as after 5-days treatment no alterations in serological markers of toxicity were observed [46]. However, there is no information about LQ-03 safety, or about the stability of any of these compounds in environmental conditions. These are important aspects to consider for the future design of sugar baits against sand flies or other insects.

The investigation of natural compounds impacting the development of *Leishmania* is a concept developed by Schlein and Jacobson for nearly three decades [17,28,29]. Over the years, this line of research has continued and expanded to other vectors, such as mosquitoes [20,24–26,69,70].

None of the drugs reduced the parasite load in sand flies infected with *Le. amazonensis*. This may be interpreted as low effectiveness, as we added the compounds to the sugar diet at a concentration ten times greater than the IC_50_ calculated for *Le. infantum* or *amazonensis,* according to Table 1. For LQB-475, we used the IC_50_ value from Table 1 for *Le. infantum*, as it was the previously established concentration. However, possibly LQB-475 was used at a concentration 35 times higher than the IC_50_ since this value for *Le. amazonensis* reported in the literature is 0.40 ± 0.06 µM [39]. Moreover, our group has made significant progress in this area; we identified that secondary plant metabolites, added to the sugar solution, were able to reduce the percentage of infected females and the parasite load in infected *Lu. longipalpis* with *L. mexicana* [27], so we expected a similar effect.

Sand flies store sugar in their crop, and the sugar passage to the digestion site (thoracic midgut) takes place slowly, according to the insect’s physiological requirement for sugar consumption [10,71–75]. Sugar feeding is more frequent in gravid females or before the next blood feed [76]. Thus, after blood digestion, females may need to replenish their energy levels with more sugar intake. At this moment, the nectomonad forms would escape from the matrix and migrate to the anterior region of the gut, a region with a higher concentration of sugar mixed with the tested compounds.

A pivotal consideration pertains to the volume ingested by females when sugar solution is mixed with different compounds. It is important to highlight that the compounds LQB-475, LQB-181, PMIC-4 and Pent had no significant effect in the volumes of sugar solution ingested by females when compared to controls, with a low effect of LQ-03 in this parameter. However, LQ-03 did not affected the preference of females for control or experimental sugar meals [47]. So, the volume of solution containing LQB-475 ingested by females was probably higher than that of solution with LQ-03. As a result, the parasites may have been exposed to different drug concentrations, unlike the observed for pentamidine; when the females possibly ingested volumes close to 60 nL.

We expected that the *in vivo* effects of these pterocarpanquinones would be closer to those observed *in vitro* since they present promastigote growth inhibition effects, alteration in mitochondrial membrane potential (ΔΨm), DNA fragmentation at dose-dependent concentrations (≤ 10 µM) and increased production of ROS, with LQB-475 having the lowest IC50 values for the promastigote forms of *Le. amazonensis*, *Le. braziliensis*, and *Le. infantum* compared to the other drugs tested, including LQB-474, LQB-182 and LQB-118 [41,42,77]. Quinone derivatives also could cause oxidative stress or even interact with DNA topoisomerases, leading *Leishmania* to death by apoptosis [78].

We also did not observe statistically significant changes in the percentage of infected females. It is important to consider that for LQ-03, the IC_50_ used was the one determined previously for *Le. infantum* and that a very different sensibility to the drug in the two parasite species can’t be discarded, as the IC_50_ for *Le. amazonensis* is still unknown.

The absence and loss of anti-*Leishmania* activity in females of *Lu. longipalpis* treated with any of the tested compounds may be related to the metabolism of these compounds by the insect. The metabolism of MSPs of several classes, including quinones, in the gut of lepidopterans has been documented [79], and mechanisms of anthraquinone degradation have been proposed in termites [80]. In this sense, it would be essential to evaluate the possible excretion of these compounds in feces and urine or even their recruitment in catabolic pathways or detoxification by the insect. Another perspective is to assess the permanence of these compounds in the female diverticulum or other organs.

Another possible mechanism of inactivation of anti-*Leishmania* activity after insect ingestion may involve intestinal microbiota’s metabolism or modification of these compounds. The action of the intestinal microbiota on MSP and its inactivation has been well documented [81], and it is possible that other microorganisms indirectly benefit the parasites. Given the potential antimicrobial properties of these compounds, continuous insect consumption might have favored the selection of drug-resistant bacteria with a high capacity to metabolize them. Thus, future studies should also consider the influence of the intestinal microbiota. In this context, it is important to consider that this study was performed with sand flies from a laboratory colony that was established in the 70’s. It is possible that these sand flies have a microbiota different from field insects’, being associated with particular microorganisms that can interfere with the relationship with the compounds tested in this work.

Another perspective is the selection of parasites resistant to the compounds during continuous exposure. The insect phase of the parasite life cycle facilitates genetic material exchange between strains, with possible diversification and selection of resistant parasites [82]. In general terms, the observed results indicate that the exposure of *Leishmania* to antiparasitic compounds in the intestine of sand flies should consider metabolic interactions between parasite, vector, and microbiota. The complexity of this biological system and the emergence of resistance phenotypes to the target compounds are limiting factors of the proposed transmission block strategy and should be the subject of future studies.

## Conclusions

We concluded that compounds LQB-181 and PMIC-4 reduced the longevity of adult sand flies and influenced the hatching of eggs, being promising candidates as lead compounds for the development of new insecticides due to their toxic effects. Compounds LQ-03 and Pent benefited insects and increased the lifespan of males and females, respectively. Compound LQB-475 reduced the longevity of *Lu. longipalpis* females, but did not decrease infection rates or parasite loads.

Our study offers some novel insights into the multifaceted impacts of compounds on insect biology, encompassing longevity, blood feeding, fecundity, fertility, and protein intake. These findings shed light on the intricate interplay between compounds and various physiological aspects, contributing to a deeper understanding of their effects on insects. The study suggests that assessing *Leishmania* exposure to antiparasitic compounds within the sand fly intestine through sugar baits necessitates the knowledge of the physiology of sugar digestion and acknowledging the potential metabolic interplay between parasite, vector, and microbiota.

## Supporting information

S1 Table Structures of the tested compounds and compositions of sugar solutions used in the experiments. Volumes of stock solutions (10 mM in DMSO) of each compound added to 1 mL of final mixture to achieve working concentrations as described in Table 1, and properties of working sugar solutions added to the sugar baits. DMSO corresponds to the group “Control 2”, and for this group the stock solution in the table below corresponds to the pure DMSO solvent.
(DOCX)

S1 File Experimental data. Raw experimental data used for statistical analysis and to make Figures 1–6.
(XLSX)
