# Supplementary material for: Evaluation of sugar meal administered anti-Leishmania compounds on the vectorial capacity of the vector, Lutzomyia longipalpis
Source: PLoS One. 2025 Jun 25;20(6):e0325178. doi: 10.1371/journal.pone.0325178 (PMC12194183; doi:10.1371/journal.pone.0325178)
Supplement: S1 Table — Volumes of stock solutions (10 mM in DMSO) of each compound added to 1 mL of final mixture to achieve working concentrations as described in Table 1, and properties of working sugar solutions added to the sugar baits. DMSO corresponds to the group “Control 2”, and for this group the stock solution in the table below corresponds to the pure DMSO solvent. (DOCX) [file pone.0325178.s001.docx]

**Supplementary Table 1.** Volumes of stock solutions (10 mM in DMSO) of each compound added to 1 mL of final mixture to achieve working concentrations as described in Table 1, and properties of working sugar solutions added to the sugar baits. DMSO corresponds to the group “Control 2”, and for this group the stock solution in the table below corresponds to the pure DMSO solvent.

| Compound name | Class | Structure | µL stock, compound | µL sucrose 70% (w/v) solution | [compound], µM | [sucrose], % (w/v) | [DMSO], % |
| --- | --- | --- | --- | --- | --- | --- | --- |
| LQB-475 | Pterocarpan-quinone |  | 1.4 | 998.6 | 14 | 69.90 | 0.14 |
| LQB-181 | Pterocarpan-quinone |  | 2 | 998 | 20 | 69.86 | 0.2 |
| LQ-03 | Pterocarpan-quinone |  | 1.7 | 998.3 | 17 | 69.88 | 0.17 |
| PMIC-4 | Hydroxyethyl-piperazine | 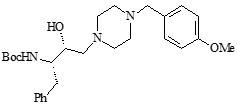 | 1.8 | 998.2 | 18 | 69.87 | 0.18 |
| Pentamidine | Aromatic-diamidine | 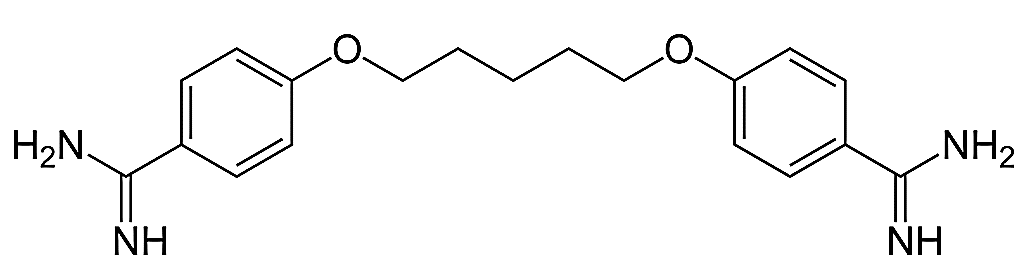 | 5.7 | 994.3 | 57 | 69.60 | 0.57 |

**Supplementary Table 1 (cont.)**

| Compound name | Class | Structure | µL stock, compound | µL sucrose 70% (w/v) solution | [compound], µM | [sucrose], % (w/v) | [DMSO], % |
| --- | --- | --- | --- | --- | --- | --- | --- |
| Amphotericin B | Polyene | 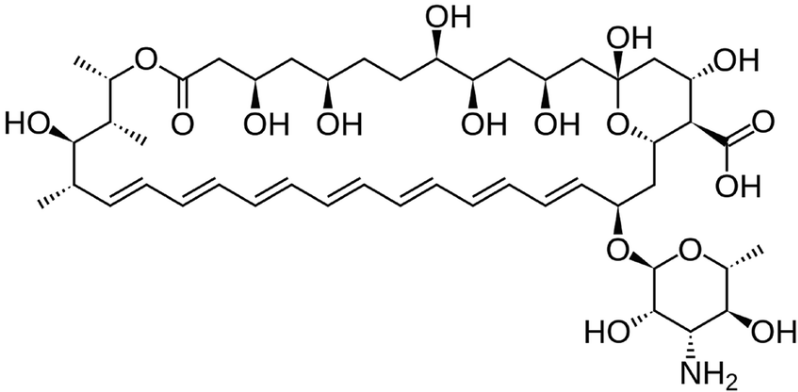 | 2.2 | 997.8 | 22 | 69.85 | 0.22 |
| DMSO | Solvent | 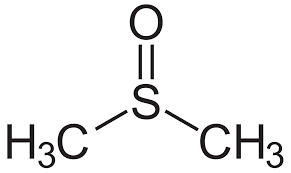 | 5.7 | 994.3 | - | 69.60 | 0.57 |
